# Centralized Measurement Level Fusion of GNSS and Inertial Sensors for Robust Positioning and Navigation

**DOI:** 10.3390/s25092804

**Published:** 2025-04-29

**Authors:** Mohamed F. Elkhalea, Hossam Hendy, Ahmed Kamel, Ashraf Abosekeen, Aboelmagd Noureldin

**Affiliations:** 1 Electrical and Computer Engineering, Military Technical College, Cairo 11766, Egypt; m.fathi@ieee.org (M.F.E.); ashraf.abosekeen@mtc.edu.eg (A.A.); 2 Electrical and Computer Engineering, Royal Military College of Canada, Kingston, ON K7K 7B4, Canada

**Keywords:** global navigation satellite system (GNSS), smartphone positioning, GNSS raw measurements, inertial navigation system (INS), GNSS/INS integration, tightly coupled integration (TC)

## Abstract

In the current era, which is characterized by increasing demand for high-precision location and navigation capabilities, various industries, including those involved in intelligent vehicle systems, logistics, augmented reality, and more, heavily rely on accurate location information to optimize processes and deliver personalized experiences. In this context, the integration of Global Navigation Satellite System (GNSS) and inertial sensor technologies in smartphones has emerged as a critical solution to meet these demands. This research paper presents an algorithm that combines a GNSS with a modified downdate algorithm (MDDA) for satellite selection and integrates inertial navigation systems (INS) in both loosely and tightly coupled configurations. The primary objective was to harness the inherent strengths of these onboard sensors for navigation in challenging environments. These algorithms were meticulously designed to enhance performance and address the limitations encountered in harsh terrain. To evaluate the effectiveness of these proposed systems, vehicular experiments were conducted under diverse GNSS observation conditions. The experimental results clearly illustrate the considerable improvements achieved by the recommended tightly coupled (TC) algorithm when integrated with MDDA, in contrast to the loosely coupled (LC) algorithm. Specifically, the TC algorithm demonstrated a remarkable reduction of over 90% in 2D position root mean square error (RMSE) and a 75% reduction in 3D position RMSE when compared to solutions utilizing the weighting matrix provided by Google with all visible satellites. These findings underscore the substantial advancements in precision resulting from the integration of GNSS and INS technologies, thereby unlocking the full potential of transformative applications in the realm of intelligent vehicle navigation.

## 1. Introduction

In the context of intelligent transportation systems, vehicle localization plays a pivotal role in enabling autonomous and semi-autonomous functionalities. Accurate localization allows vehicles to determine their position with high precision, which is essential for safe navigation, decision-making, and interaction with dynamic environments. As intelligent vehicles become increasingly integrated with advanced perception and control systems, the demand for robust and reliable localization methods continues to grow [1,2].

Localization plays a critical role in various aspects of our daily lives, from aiding vehicle rescue operations to simplifying parking and enhancing navigation. Beyond these practical applications, localization data have taken on a pivotal role in the broader landscape of the Internet of Things (IoT). In scenarios where human presence or direct involvement may be limited, the provision of precise location information to IoT devices has become indispensable. There are two basic ways to obtain a navigation solution: dead reckoning (DR) and position fixing [3,4]. DR is a navigation method that estimates position based on prior knowledge of the initial location, heading, and velocity. This principle is analogous to the operational concept of an inertial navigation system (INS) in autonomous mode, where position updates are derived from initial conditions and the continuous integration of inertial sensor outputs. In contrast, position fixing, where the location is derived from external references such as Global Navigation Satellite Systems (GNSSs), serves as an excellent example. The navigation system can be independent, rely on external sources, or combine both. A Global Positioning System (GPS) requires the assistance of other sources, such as cellular base stations, motion sensors such as (compass, inertial sensors, odometer), Wi-Fi, aiding sensors such as Radar, LIDAR and cameras, and digital maps to enhance localization [5,6].

Among the numerous navigation techniques available, satellite navigation and inertial navigation are the most common. It is possible to unlock new levels of connectivity and location-based services in the IoT landscape by integrating GNSS/INS capabilities into smartphones. This will have a significant impact on a variety of businesses as well as our daily lives. The IoT gains an essential layer of spatial awareness thanks to the inclusion of GNSS and INS in smartphones. Smartphones can determine their precise position on the Earth’s surface using GNSS technology, which offers accurate Position, Velocity, and Time (PVT) solutions through satellite constellations such as GPS, GLONASS, Galileo, and BeiDou. However, standard GNSS receivers are not capable of independently determining device orientation. To overcome this limitation, smartphones integrate GNSS with INS, which utilize onboard sensors—such as accelerometers, gyroscopes, and, sometimes, magnetometers—to track motion and orientation. Additionally, orientation information can be further enhanced through the use of multi-antenna GNSS configurations or other complementary sensors [3]. This fusion enables smartphones to provide robust navigation capabilities, including not only position and velocity but also reliable orientation estimation [2,7]. The seamless integration of GNSS and INS technologies in smartphones not only extends the capabilities of the IoT but also brings new dimensions to the world of intelligent vehicles.

1. It allows for the continuous surveillance and monitoring of assets and resources. This integration can be used to optimize operations, increase efficiency, and reduce costs in industries such as logistics, supply chain management, and transportation. Companies, for example, may properly track shipments in real-time, monitor routes, and ensure timely deliveries by equipping delivery vehicles with cellphones equipped with a GNSS/INS [2].
2. Context-aware services and personalized experiences are made possible by the integration of GNSS/INS in smartphones. Location-based applications can employ exact positioning data to deliver personalized suggestions, local information, and targeted adverts to users. This technology supports smart city projects by providing real-time information on transport, parking availability, surrounding amenities, and cultural events to city people, encouraging a more connected and comfortable urban lifestyle [8,9].
3. It improves the safety and security of the IoT ecosystem. Emergency response systems can quickly detect and assist persons in need during critical situations by correctly determining the position and motions of humans and assets. In addition, IoT-enabled security systems can benefit from smartphones with a GNSS/INS, which provide exact geolocation for tracing stolen devices, guarding restricted areas, and assuring human safety [10].

There are three various types of integration possible between the two systems to improve navigation data; loosely coupled (LC), tightly coupled (TC), and ultra-tightly coupled integration [11,12,13,14,15]. Over the past decade, the positioning techniques of smartphones and their ability to navigate using GNSSs have advanced significantly [16]. Nowadays, smartphones are equipped with more than 25 sensors, making it possible to use these sensors like gyroscopes and accelerometers to create a complete INS [3,17]. Google announced in May 2016 access to raw GNSS measurements (RGNSSM) on all Android devices operating with Nougat Android (7.0) and higher [18]. RGNSSM processing enables new localization and navigation methods, one of which involves integrating smartphone GNSS technology with an INS [16,19]. LC integration is the simplest integration technique with the least computational time. However, there are two Kalman Filters (KFs) used, one for the GNSS and the other for integration, which may cause cascaded filtering problems. Furthermore, as the number of available satellites decreases, the accuracy of the INS prediction degrades. Once the satellite count drops below four, the solution can no longer be updated using GNSS measurements. Such problems can be solved smoothly by the TC integration technique, which is called centralized integration and has a common master filter and GNSS pseudorange and pseudorange rate, and corresponding INS predicted values are used in the master KF to determine the accurate integrated solution. Therefore, the INS can use the remaining satellites, even if the number falls below four satellites, to continuously update its measurements [3,20].

### 1.1. Objectives

This study aimed to enhance the precision of smartphone positioning by adopting the approach elucidated in [19]. For ease of reference, we will refer to this approach as the Modified Downdate Algorithm (MDDA). The algorithm was precisely designed to select the most optimal visible satellites that are positioned above the horizon. Additionally, it introduces a distinctive weighted matrix aimed at augmenting the precision of smartphone low-cost GNSS receivers. Furthermore, the effectiveness of the new algorithm was evaluated when combined with a low-cost smartphone Inertial Measurement Unit (IMU) using both LC and TC integration techniques, considering the motion dynamics. To validate the proposed method, real road trajectory data collected from low-cost smartphone sensors were utilized. The main contributions of this study are as follows:

1. The application of satellite selection-based MDDA to both LC and TC algorithms for all visible satellites.
2. The application of the proposed algorithm in scenarios where the receiver is limited to a specific number of channels.
3. The analysis of the impact of the selected satellite-based MDDA on the overall positioning performance.

These contributions aim to optimize smartphone positioning accuracy by improving the satellite selection process and investigating the influence of selected satellite-based MDDA on the performance of LC and TC integration techniques. Additionally, the performance of the TC method during various simulated outage periods with different visible satellites at this outage is analyzed.

### 1.2. Paper Organization

This paper is structured as follows: Section 2 outlines the related research, Section 3 details the approach, Section 4 elaborates on the experimental setup, and Section 5 discusses the analysis and results. The conclusion of this study is presented in Section 6.

## 2. Related Work

Numerous investigations have been undertaken to assess and augment the localization accuracy of smartphones. Nevertheless, it is important to highlight that a significant portion of these studies have primarily concentrated on scrutinizing performance in optimal open-sky conditions, thereby neglecting the inherent reality that real-world smartphone positioning activities are predominantly executed within the intricate and demanding contexts of urban environments, where formidable challenges and adverse conditions persist [21,22,23,24,25].

In [2], an approach was introduced to enhance the integration of GNSS/INS and visual inertial navigation systems (VINSs). This approach involved the application of the Semantic Proximity Update (SPU) technique, making use of a pre-trained model for real-time object detection. SPU entails the identification of geo-referenced objects along with tracking their relative movement to ascertain an absolute position. The empirical findings indicated that the proposed method yielded significantly reduced errors in horizontal and three-dimensional positioning, with respective decreases of 51.6% and 86.8% compared to a conventional integration technique.

In [7], a TC multi-GNSS precise point positioning (PPP)/INS/LiDAR integrated system was designed to harness the complementary capabilities of these onboard technologies for urban navigation. In order to enhance navigation accuracy and computational efficiency, a novel LiDAR sliding-window plane-feature tracking approach was developed. The proposed integration of GNSS/INS/LiDAR demonstrated the ability to achieve submeter-level horizontal positioning accuracy in challenging GNSS environments, yielding substantial improvements of 73.3% in the east direction, 59.7% in the north direction, and 64.2% in the up direction (ENU) when compared to conventional GNSS/INS integration methods.

In [12], two multi-constellation smartphone models were used. A The Samsung Galaxy S21+ (Samsung Electronics, Suwon, Republic of Korea) and the Xiaomi Mi 8 (Xiaomi Corporation, Beijing, China) were attached to a vehicle dashboard. The total root mean square error (RMSE) could be improved to less than 2 m by TC integrating PPP with the smartphone IMU in ideal conditions.

In [26], digital surface models (DSMs) pertaining to diverse urban scenarios were simulated. In urban canyon environments, a considerable portion of epochs failed to provide a minimum of four visible satellites, and numerous epochs exhibited suboptimal position dilution of precision (PDOP). Simulations were conducted to explore various geometric configurations of 5G base stations surrounding the observer. An integration between 5G service and GNSS improved the redundancy and the quality of the solutions by using five 5G base stations around the observer.

In [27], the LC algorithm was used with an iPhone 4 (Apple Inc., Cupertino, CA, USA) as a platform in a car dashboard, and the findings showed that under real GPS signal conditions, the RMSE could reach 2.07 m in the horizontal and 2.19 m in the vertical plane. This RMSE increased to 70.2 m horizontally and 16.5 m vertically within 30 s of GPS outage and decreased to 33.2 m horizontally and 15.7 m vertically after using non-holonomic constraints.

In [28], with a Huawei Mate 8 (Huawei Technologies Co., Ltd., Shenzhen, China) smartphone inside a vehicle, two scenarios were examined the first using the smartphone IMU only, reaching 2000 m to the east and 100 to the north. Only 45 s of data and 10 s of GPS outage were used during the test. Using LC integration between the phone sensors and the GPS, the error was decreased to 11 m, as mentioned during the outage.

In [29], the performance of the Xiaomi Mi 8 smartphone, equipped with dual-band capabilities, in conjunction with a geodetic receiver was examined. Specifically, the study focused on investigating the influence of multipath effects on positioning accuracy. The results indicated that the Galileo E5 frequency exhibited a horizontal RMSE of 4.57 m, while the L1/E1 multi-constellation frequency demonstrated a 2D RMSE of 5.36 m.

In [30], the authors evaluated the quality of contemporary Android smartphones’ multi-GNSS observations Xiaomi MI 8, 9 (Xiaomi Corporation, Beijing, China), and Huawei P20, P30 (Huawei Technologies Co., Ltd., Shenzhen, China)). Signal strength, observational noise, and satellite tracking capabilities were all included in the study. The positioning experiment demonstrated that it was possible to solve a smartphone positioning problem precisely at the cm level even when there were fixed integer ambiguities.

In [31], the analysis commenced with an examination of methodologies that solely relied on data acquired from vehicle on-board sensors. It demonstrated that while certain approaches can attain the requisite accuracy for autonomous driving, they encounter constraints imposed by the substantial cost of the sensors and performance limitations encountered in diverse driving scenarios, including but not limited to cornering, intersections, and challenging environmental conditions such as low light or snowy conditions. In view of this analysis, the prospect of developing cost-effective localization systems exhibiting robustness and high accuracy arose through the fusion of off-board information with sensory input. However, it is crucial to acknowledge that the efficacy of such systems is contingent upon factors such as the infrastructure availability or penetration rate of neighboring connected vehicles, as well as the level of network service.

In [32], an algorithm named “The Whole Work” was introduced, which involved tightly coupling GNSS and IMU within a filter for robust GNSS positioning with local ground augmentation gtrategies. The Unscented Kalman Filter (UKF) was employed as a primary estimator. The recorded data were obtained from a Samsung S20 Ultra device, utilizing code observations and carrier-phase measurements. The application of this algorithm resulted in improvements in horizontal accuracy. Specifically, the d50 accuracy was enhanced by 6.4%, the d80 accuracy by 5.0%, and the d95 accuracy by 18.1% when using the combination of pseudorange and Doppler measurements. d50 represented the radius of the circle within which 50% of the horizontal position errors fell. A smaller d50 value indicated higher accuracy, meaning that more of the position estimates were closer to the true location. The author used d50, along with d80 and d95, to provide a comprehensive evaluation of the distribution of errors and better illustrate the performance of the algorithm across different probability levels. Furthermore, when integrating pseudorange, Doppler, and phase measurements, the d50 accuracy was improved by 8.6%, the d80 accuracy by 8.3%, and the d95 accuracy by 19.8%.

In [33], a literature review highlighted the limitations of traditional GNSS/INS integration methods, such as the Extended Kalman Filter (EKF) and the Robust Kalman Filter (RKF), particularly in urban environments characterized by frequent signal obstructions and measurement noise. In addressing these challenges, the Factor Graph Optimization (FGO) method has shown superior robustness and accuracy. In high-precision inertial navigation experiments, the FGO approach yielded improvements of up to 81.1% in position, 69.8% in velocity, and 75.1% in attitude estimation compared to EKF and RKF. Even in consumer-grade inertial systems, the FGO model achieved up to 36.4% improvement in position, 73.8% in velocity, and 62.3% in attitude, demonstrating its effectiveness across varying sensor qualities.

In [34], a comprehensive analysis of double-differenced GNSS observations from GPS, GLONASS, and Galileo systems revealed significant variability in noise characteristics across constellations and signal types. Utilizing autocorrelation functions, Lomb–Scargle periodograms, and modified Allan deviation metrics, the study showed that GPS C5Q and Galileo C7Q/C8Q code signals exhibited the lowest noise levels (≤10 cm standard deviation), whereas GLONASS C1C and C2C signals demonstrated the highest (up to 90cm). Carrier phase observations were notably consistent across all systems, with noise levels ranging from 1.5 to 3.5mm. Temporal correlation analysis indicated that 1 Hz carrier phase data could be considered temporally uncorrelated, unlike code observations, which required intervals exceeding 20s to achieve temporal independence. Furthermore, noise types were classified as flicker or white phase modulation (PM) for code signals, while GPS and Galileo carrier phases were exclusively characterized by white PM.

## 3. Methodology

The proposed methodologywas structured around three core components designed to enhance positioning accuracy. The first component, represented by the proposed MDDA algorithm, addresses the GNSS receiver level by optimizing satellite selection through an appropriate weighting matrix and applying satellite exclusion strategies when necessary.

The second component focuses on the inertial navigation system (INS), where accuracy is improved through precise calibration techniques aimed at minimizing inertial sensor errors.

**Figure 1:**
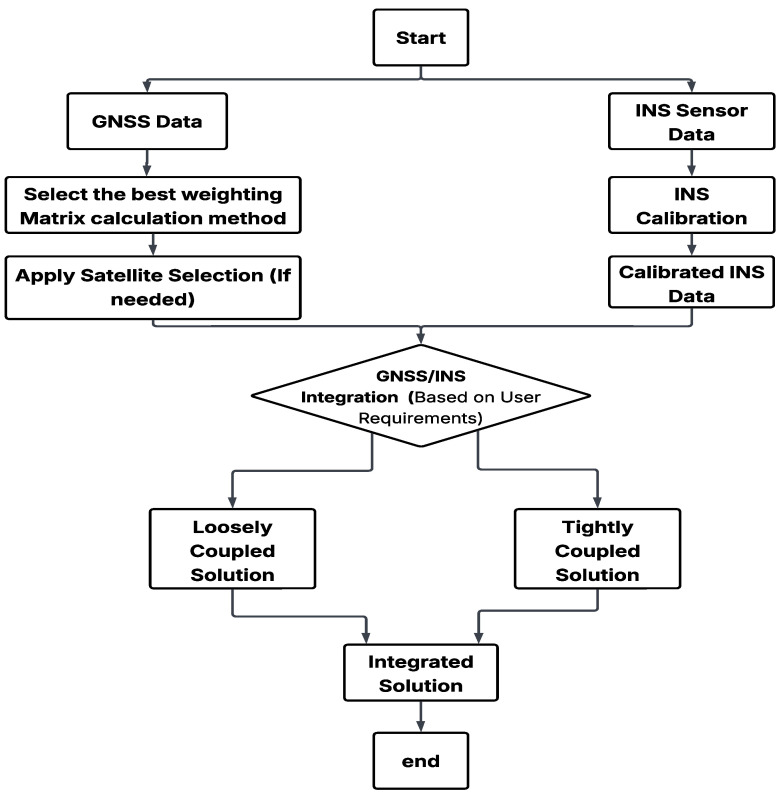
Flowchart of the main methodological components.

The third component involves the integration of refined GNSS and INS data using two fusion strategies: loosely coupled (LC) and tightly coupled (TC), as illustrated in Figure 1. Each of these components is discussed in detail in the following sections.

### 3.1. Data Recording and Analysis

Google has recently unveiled a set of valuable resources aimed at fostering developer engagement with RGNSSM. These resources include the GNSS Logger v3.0.6.4, MATLAB Mobile v6.3.0, and GPS Test v3.10.3 applications, which are conveniently available on the Android application store, enabling developers to efficiently gather data for analysis. Subsequently, Windows-based programs such as Emlid Studio and GNSS Analysis v4.6.0.1 were employed to analyze the data acquired during the initial stage.

### 3.2. Coordinate Frames

**Figure 2:**
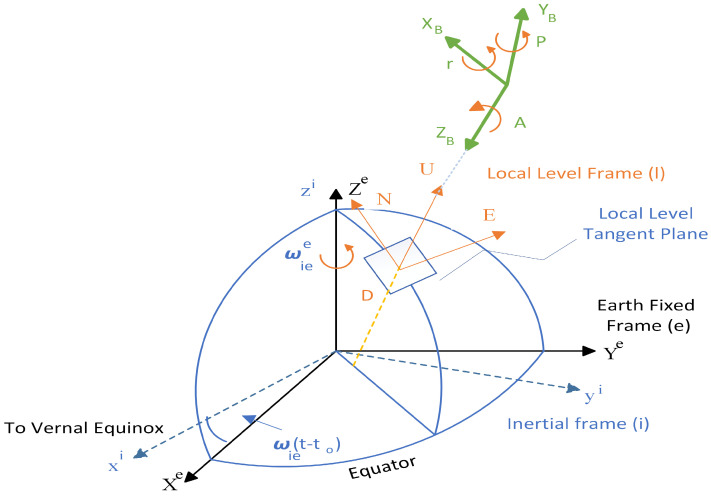
The coordinate frames.

The transformation of quantities, encompassing parameters such as attitude, velocity, and position, across diverse coordinate frames is an essential requirement for any navigation system. In this research, four specific coordinate frames were utilized: the Earth-centered Earth-fixed (ECEF), local level (LLF), body (B), and Earth-centered inertial (ECI) frames. The coordinate frames utilized in this paper are illustrated in Figure 2 [3].

### 3.3. Traditional Weighting Matrix Model

In the literature, numerous weighting models rely on either the satellite’s elevation (EL), carrier-to-noise density ($C/N_{o}$), or a combination of both these parameters to give a weight for each satellite signal. Additionally, this step acts as an input to the satellite selection/rejection techniques. An overview of the traditional weighting matrix models is explained as follows:

1. Elevation-based model (EL model): In this particular model, the variance, carrier-phase observation model is dependent on the satellite’s elevation angle (EL) and utilizes mathematical functions such as $\sin^{2}\left(EL\right)$ or sin(EL) [35,36,37]:
  (1) $$\sigma_{Pr}^{2}=\frac{\sigma_{o}^{2}}{\sin^{2}\left(EL\right)}$$
  (2) $$\sigma_{Pr}^{2}=\frac{\sigma_{o}^{2}}{\sin(EL)}$$
  where $\sigma_{o}^{2}$ is the error variance of the pseudorange.
2. Carrier to noise density-based model ($C/N_{o}$ model): In [35,36], the model was structured around the signal-to-noise ratio $C/N_{o}$, expressed as follows:
  (3) $$\sigma_{Pr}^{2}=b\cdot 10^{\frac{-C/N_{0}}{10}}$$
  where b is constantly defined empirically and equals $10^{4}\;m^{2}$. In [12,38,39], the proposed standard deviation of code observation
  (4) $$\sigma_{pr}=a+b\;10^{\frac{-C/N_{o}}{20}}$$
  In this context, the coefficient *a* represents the root mean square value (RMS) of pseudorange observation residuals, while *b* corresponds to the pseudorange coarse acquisition (C/A-code) chipping length, which was defined as 293 m for L1 measurements.
3. $EL-C/N_{0}$-model: In [35,37,40,41], a combination model was proposed that depended on both El and $C/N_{o}$:
  (5) $$\sigma_{pr}^{2}=k\;\frac{10^{\frac{-C/N_{o}}{10}}}{\sin^{2}El}$$
  where *k* is 1 if the line-of-sight (LOS) signal is available and 2 or *∞* if no LOS signal is available:
  (6) $$\sigma_{pr}^{2}=x+y\;\frac{10^{\frac{-C/N_{o}}{10}}}{\sin El}$$
  (7) $$\sigma_{pr}^{2}=x+\frac{y}{\sin El}+z\;10^{\frac{-C/N_{o}}{10}}$$
  where x, y, and z model a nonlinear regression problem that relates the mean of Inverse-Gamma estimated distributions and the corresponding $EL-C/N_{o}$ pair [40].
4. Google proposed sigma: In the smartphone’s RGNSSM, there is a provided value referred to as Received Space Vehicle Time Uncertainty Nanos (RSVTUN). This value signifies the estimated discrepancy in the received GNSS time.
  (8) $$\sigma_{pr}^{2}=\mathrm{RSVTUN}\times 10^{9}\times c$$
  Google advises filling the weighting matrix with this value, yet neither the Android developer’s guide nor the accompanying white paper offers guidance on its calculation [16].

### 3.4. Satellite Selection (SS) Techniques

In this subsection, various methods are explored for eliminating noisy measurement satellites or, in other words, determining the set of satellites used for positioning.

1. Based on El: This model is employed by selecting the satellite with the highest (EL) angle and disregarding the rest. While this approach ensures good traceability, it may not provide the best availability [42,43].
2. Based on $C/N_{0}$: In [44], a fuzzy SS algorithm was utilized to select a set of satellites with the highest $C/N_{0}$ and minimum geometrical dilution of precision (GDOP).
3. Brute force (optimal) method: Through the process of evaluating all possible combinations and selecting the optimal performance set, this approach ensures the best performance. However, it comes with the drawback of high computational cost [43].
  Given *n* satellites on the horizon and a receiver with *k* channels, the set of available options for the optimal method, denoted as $AS_{\mathrm{opt}}$, is determined by
  (9) $$AS_{opt}=\frac{n!}{(n-k)!k!}$$
  For example, if n = 30 and k = 25, 142,506 geometries should be evaluated.
4. Greedy method: Similar to the optimal method for evaluating subset performance, this approach removes only one satellite at a time and utilizes the determined geometry to calculate the next iteration [43,45].
  The numbers of $AS_{greedy}$ are given by
  (10) $$AS_{greedy}=\frac{1}{2}\left[n\left(n+1\right)-k\left(k+1\right)\right]$$
  For example, if n = 30 and k = 25, then the number of geometries to evaluate is reduced to 140.
5. Downdate method: Given our objective to optimize elements of the covariance matrix, we revert to employing the greedy algorithm approach. The position covariance matrix (CM) in the north–east–down (NED) frame is used:
  (11) $$\mathrm{CM}={\left(G^{T}RG\right)}^{-1}$$
  where G is the geometry matrix in the NED frame and R is the weighting matrix.
  To find the best subset, the CM must be calculated in Equation (12):
  (12) $${\mathrm{CM}}_{i}=\mathrm{CM}+\frac{S_{i}.S_{i}^{T}}{P_{i,i}}$$
  where $CM_{i}$ is the position CM with the $i^{th}$ removed satellite and $S_{i}$ is the $i^{th}$ *S* matrix column.
  (13) $$S={\left(G^{T}RG\right)}^{-1}G^{T}R$$
  (14) $$P=R-RG{\left(G^{T}RG\right)}^{-1}G^{T}R$$
  Although the downdate method suggests a more efficient way to implement the greedy algorithm, it also provides insights into an even more efficient algorithm. By examining Equation (12), we can observe this potential improvement.
  (15) $${\left({\mathrm{CM}}_{j,j}\right)}_{i}={\mathrm{CM}}_{j,j}+\frac{S_{j,i}^{2}}{P_{i,i}}$$
  The rise in CM will manifest in the final term of Equation (15). The smaller this term, the less impact it will have on increasing the corresponding covariation term.
  To obtain the best subset, one can sort the values in Equation (16) from all calculations and then retain the largest values of this cost function [43,46].
  (16) $$\mathrm{cost}\;\mathrm{function}=\frac{0.25\left(S_{1,i}^{2}+S_{2,i}^{2}\right)+S_{3,i}^{2}}{P_{i,i}}$$
  This method outperforms the greedy approach. Similar to the EL-based method, it involves calculating a set of all in-view solutions initially and then selecting the optimal subset.

### 3.5. The Proposed MDDA Algorithm

The proposed MDDA algorithm introduces a distinctive weighted matrix designed to enhance the accuracy of low-cost GNSS receivers in smartphones. This weighting matrix is then integrated into the downdate SS algorithm to provide the best set of satellites that can be utilized in better positioning calculations.

The proposed weighting matrix is constructed by combining Equations (1), (4) and (6) to factor in the influence of both satellite elevation (El) and carrier-to-noise ratio ($C/N_{0}$). This integration is expressed as follows:

(17) $$\sigma_{Pr}^{2}=k+cl\left(\frac{10^{\frac{-C/N_{0}}{10}}}{\sin^{2}\left(El\right)}\right)$$

where $\sigma_{Pr}^{2}$ is the proposed variance value, *k* represents the RMS of pseudorange observation residuals, while cl corresponds to the chipping length of pseudorange-coarse-acquisition (C/A-code), which is defined as 293 m for L1 measurements, based on standard GNSS specifications that optimize signal processing. Moreover, the weighting matrix *R* is formulated as follows:

(18) $$R=diag[{\sigma_{Pr}^{2}}_{1}\;{\sigma_{Pr}^{2}}_{2}\;\ldots \;{\sigma_{Pr}^{2}}_{m}]$$

where ${\sigma_{Pr}^{2}}_{1:m}$ is the proposed variance value from satellite 1 to satellite m.

Additionally, this weighting matrix is integrated with the downdate SS algorithm to filter out satellites with noisy measurements. The selection process entails picking satellites that demonstrate both 3D position RMSE values lower than the average of the $C/N_{0}$ values on the horizon and elevation angles below a designated threshold. Moreover, in situations where the receiver has a restricted number of channels, the MDDA selects the most optimal satellite while disregarding the rest.

Assessing the efficacy of the MDDA algorithm, it transcends its exclusive application with a GNSS receiver. Furthermore, its effectiveness is observed when incorporated into the integration framework of a GNSS receiver and an inertial navigation system, encompassing both loosely coupled and tightly coupled integration schemes.

### 3.6. MDDA Augmented GNSS/INS Integration

In a GNSS/INS fusion, IMU usually provides a higher data rate than the GNSS receivers. In our case, the GNSS receiver sampling rate was 1 Hz, with 100 Hz for the IMU. In this study, two integration models were employed to combine the RGNSSM with data from smartphone inertial sensors, and these models were further enhanced by the proposed MDDA.

**Figure 3:**
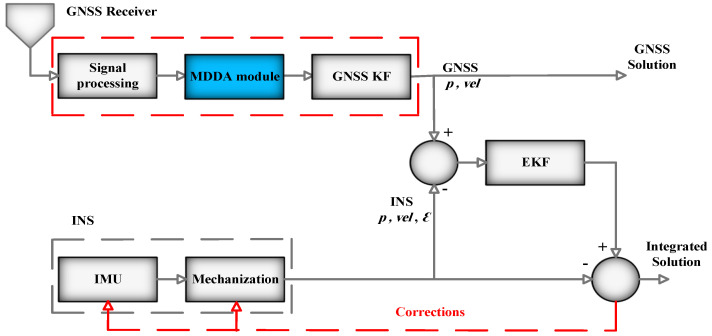
LC integration architecture.

The architecture for LC integration, incorporating the MDDA module, is visually depicted in Figure 3. In this LC configuration, the module was positioned before the GNSS Kalman Filter (KF) to select the most suitable subset of satellite measurement. This subset is then used to compute the GNSS receiver’s position and velocity.

**Figure 4:**
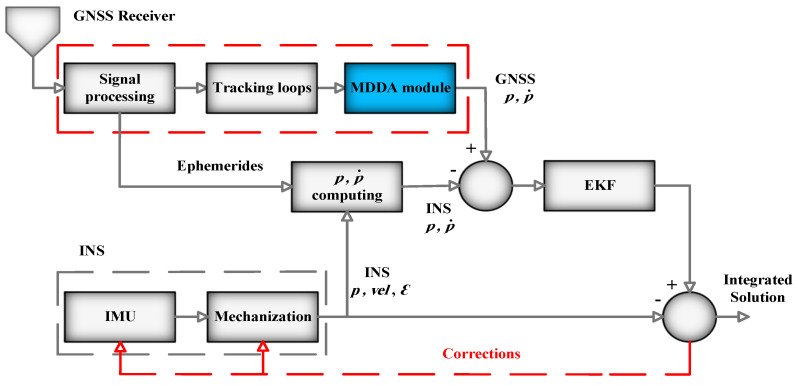
TC integration architecture.

Similarly, in Figure 4 for TC integration, the module is placed after the tracking loop. It performs the task of selecting the optimal subset, which includes pseudorange and pseudorange rate data. The subsequent procedural step descriptions will elucidate these processes in greater detail.

#### 3.6.1. IMU Calibration

In the realm of smartphone sensors, calibration holds paramount importance as it facilitates the computation of deterministic errors. The process of calibration entails assessing sensor errors in a controlled laboratory environment. The calibration requirements may vary depending on the specific technology employed in an IMU. The precise determination of all parameters necessitates the use of specialized calibration instruments, such as three-axial turntables, to conduct a six-position static test. In [11], calibration was carried out specifically for the gyroscope (gyro) and accelerometer (acc) of the smartphone. This study primarily focused on investigating the impact of scale factor and bias, two prominent sources of deterministic errors, on the IMU solution and the resulting position error dx as follows:

(19) $$dx_{acc}≅\;\frac{1}{2}\;b_{acc}\;t^{2}$$

(20) $$dx_{gyro}≅\;\frac{1}{6}\;b_{gyro}g\;t^{3}$$

where $b_{acc}$ and $b_{gyro}$ are biases in the acc and gyro, which are responsible for the position errors $dx_{acc}$ and $dx_{gyro}$, respectively. *t* is the mechanization time of IMU and *g* is the Earth’s gravity. In other words, as shown in Equations (19) and (20), this bias introduces a second-order and third-order error in position, respectively [3,11,28,47].

Table 1 presents the specification and deterministic error of the BMI160 BOSCH gyro and acc, which were installed on the REALME RMX2030 smartphone [48].

#### 3.6.2. INS Mechanization

Local level frame (LLF) mechanization equations have been requested in many applications because the user may easily understand the navigation solution provided by the LLF navigation equations when they are on or near the surface of the Earth [49]. In the ECEF frame, the position vector $p^{l}$ is defined as shown in Equation (21):

(21) $$p^{l}=\left[\begin{array}{c} \varphi \\ \lambda \\ alt \end{array}\right]$$

where λ is the longitude long, φ is latitude lat, and alt is altitude. The velocity components influence the temporal rate of variation of the position components [3]:

(22) $$\left[\begin{array}{c} \dot{\varphi }\\ \dot{\lambda }\\ \dot{alt} \end{array}\right]=\left[\begin{array}{ccc} \frac{1}{R_{m}+alt} & 0 & 0\\ 0 & \frac{1}{\cos\phi (R_{n}+alt)} & 0\\ 0 & 0 & -1 \end{array}\right]{\left[\begin{array}{c} vel_{n}\\ vel_{e}\\ vel_{d} \end{array}\right]}^{l}$$

(23) $${\dot{p}}^{l}=A^{-1}vel^{l}$$

where $\dot{\lambda }$, $\dot{\varphi }$, and $\dot{a}lt$ are temporal rate of variations of long, lat, and alt. $vel_{n}$, $vel_{e}$, and $vel_{d}$ are the velocities in the north, east, and down (NED) directions, respectively. $R_{n}$ and $R_{m}$ are normal and meridian ellipsoid radii. $A^{-1}$ is the transformation matrix of the velocity vector between rectangular and curvilinear coordinates.

The time derivative of LLF velocity component $vel^{l}$ is shown in Equation (24).

(24) $$vel^{l}=C_{b}^{l}ax^{b}-\left(2\Omega_{ie}^{l}+\Omega_{el}^{l}\right)vel^{l}+g^{l}$$

where the $C_{b}^{l}$ matrix transforms the b-frame to the l-frame, $ax^{b}$ is the accelerometer’s measurement in the b-frame, $g^{l}$ is the vector of gravity in the l-frame, and $\Omega_{ie}^{l},\;\Omega_{el}^{l}$ are the skew–symmetric matrices related to $\omega_{ie}^{l},\;\omega_{el}^{l}$, which are Earth rotation rate and transportation rate [3].

The orientation (attitude) is calculated by solving the transformation matrix’s time derivative equation ${\dot{C}}_{b}^{l}$ as follows:

(25) $${\dot{C}}_{b}^{l}=C_{b}^{l}\left[\Omega_{ib}^{b}-C_{l}^{b}\;\left(\Omega_{ie}^{l}+\Omega_{el}^{l}\right)C_{b}^{l}\right]$$

where $\Omega_{ib}^{b}$ is the skew–symmetric matrix of the gyro-measurement vector. The quaternion was used for the following reasons [3,49,50]:

1. The singularity issue that can occur when using Euler angles is avoided by the quaternion solution.
2. It is rather easy to execute the quaternion computation.
3. Instead of six differential equations if directions cosines are used, just four equations are numerically solved.

Quaternion *q* has four parameters $(q_{1},q_{2},q_{3}$, and $q_{4})$ and is represented as follows:

(26) $$q={\left[\begin{array}{c} \cos\left(\frac{\theta }{2}\right),\;\left(\frac{\theta_{x}}{\theta }\right)\sin\left(\frac{\theta }{2}\right),\;\left(\frac{\theta_{y}}{\theta }\right)\sin\left(\frac{\theta }{2}\right),\;\left(\frac{\theta_{z}}{\theta }\right)\sin\left(\frac{\theta }{2}\right) \end{array}\right]}^{T}$$

where θ is the rotational angle of the quaternion and equals $\sqrt{\left(\theta_{x}^{2}+\theta_{y}^{2}+\theta_{z}^{2}\right)}$ and $\frac{\theta_{x}}{\theta },\frac{\theta_{y}}{\theta },\frac{\theta_{z}}{\theta }$ is the directional cosine of the rotation axis. Additionally, quaternion obeys the unity restriction where $1={q_{1}}^{2}+{q_{2}}^{2}+{q_{3}}^{2}+{q_{4}}^{2}$ Hence,

(27) $$\dot{q}=\frac{1}{2}\left[\bar{\Omega }\left(\omega \right)\right]q$$

(28) $$\bar{\Omega }\left(\omega \right)=\left[\begin{array}{cccc} 0 & \omega_{z} & -\omega_{y} & \omega_{x}\\ -\omega_{z} & 0 & \omega_{x} & \omega_{y}\\ \omega_{y} & -\omega_{x} & 0 & \omega_{z}\\ -\omega_{x} & -\omega_{y} & -\omega_{z} & 0 \end{array}\right]$$

where $\omega ={\left[\omega_{x}\;\omega_{y}\;\omega_{z}\right]}^{T}$ is the body rotation angular velocity.

The transformation matrix from the body frame to the LLF using the direction cosine matrix and quaternion states is illustrated by (29) [49,51,52].

(29) $$\begin{array}{c} C_{b}^{l}=\left[\begin{array}{ccc} q_{2}^{2}+q_{1}^{2}-q_{3}^{2}-q_{4}^{2} & 2(q_{2}q_{3}-q_{4}q_{1}) & 2(q_{3}q_{4}+q_{3}q_{1})\\ 2(q_{2}q_{3}+q_{4}q_{1}) & q_{3}^{2}+q_{1}^{2}-q_{2}^{2}-q_{4}^{2} & 2(q_{3}q_{4}-q_{2}q_{1})\\ 2(q_{2}q_{4}-q_{3}q_{1}) & 2(q_{3}q_{4}+q_{2}q_{1}) & q_{4}^{2}+q_{1}^{2}-q_{2}^{2}-q_{3}^{2} \end{array}\right] \end{array}$$

The attitude angles $\varepsilon_{3\times 1}^{l}$ can be demonstrated as follows:

(30) $$\varepsilon_{3\times 1}^{l}=\left[\begin{array}{c} \phi \\ \theta \\ \psi \end{array}\right]=\left[\begin{array}{c} -\mathrm{atan}2({C_{b}^{l}}_{31},{C_{b}^{l}}_{33})\\ \mathrm{asin}({C_{b}^{l}}_{32})\\ -\mathrm{atan}2({C_{b}^{l}}_{12},{C_{b}^{l}}_{22}) \end{array}\right]$$

#### 3.6.3. Extended Kalman Filter (EKF)

The EKF is a Kalman Filter modification that manages nonlinear systems’ state estimation [49,50]. A nonlinear system is linearized by the EKF with respect to its current states covariance and mean; hence, the state errors can represent the linear system (rather than the system’s states), which thus permits the use of the linear KF. The KF makes the assumption that each state’s time derivative is a function that is linear with the others and the white noise sources [49]. The first-order Taylor series approximation is applied to derive the linearized error-state model $\delta \dot{x}\left(t\right)$, expressed as

(31) $$\delta \dot{x}\left(t\right)=F\left(t\right)\delta x\left(t\right)+G\left(t\right)w\left(t\right)$$

where $\delta x=x-\hat{x}$ represents the estimation error between the true state vector *x* and its estimate $\hat{x}$ and consists of the position errors, velocity errors, attitude errors, and errors in the inertial sensors. Here, w(t) denotes zero-mean, unit-variance white, Gaussian-process noise; G(t) is the noise distribution matrix; and F(t) is the system dynamics matrix.

The measurement model is similarly linearized as

(32) δz(t)=H(t)δx(t)+v(t)

where H(t) is the measurement matrix, determined based on known system parameters, and v(t) is the measurement noise vector, assumed to be zero-mean white Gaussian noise.

#### 3.6.4. Error Model

Inertial sensor error is one of the main causes of process noise error.

The error model is defined as in (33):

(33) $$u_{measured}\left(t\right)=u\left(t\right)\left(1+s\left(t\right)\right)+b\left(t\right)+w\left(t\right)$$

where u(t) is the sensor input, s(t) is the scale factor error, b(t) is the bias, w(t) is random walk noise, and the measured value of the sensor is $u_{measured}\left(t\right)$. The stochastic sensor’s errors like scale factor and bias are frequently modeled using the first-order Gauss–Markov (GM) process as follows:

(34) $$\dot{n}\left(t\right)=-\beta n\left(t\right)+\sqrt{\left(2\beta \alpha^{2}\right)}w_{u}\left(t\right)$$

where n(t) is a random process, $w_{u}\left(t\right)$ a unity variance zero-mean Gaussian noise, β is correlation time inverse, and $\alpha^{2}$ is the white noise variance. By analyzing the sensor measurements over a fixed length of time, these parameters can be roughly determined [50,53].

#### 3.6.5. LC Integration Model

As shown in Figure 3, after choosing the best set of the satellite by the MDDA, the receiver can determine the position and the velocity LC integration fuses the final six-element results of the GNSS receiver position and velocity with the INS navigation solution.

The state vector is defined as

(35) $$\delta x_{15\times 1}^{l}={\left[\delta p_{3\times 1}^{l}\;\delta vel_{3\times 1}^{l}\;\delta \varepsilon_{3\times 1}^{l}\;\delta \omega_{3\times 1}\;\delta ax_{3\times 1}\right]}^{T}$$

where $\delta p_{3\times 1}^{l}$ is the error vector of position, $\delta vel_{3\times 1}^{l}$ is the error vector of velocity, $\varepsilon_{3\times 1}^{l}$ is the attitude error vector, $\delta \omega_{3\times 1}$ is the gyroscope drift error vector, and $\delta ax_{3\times 1}$ is the accelerometer bias error vector. The measurement vector of the LC model contains the difference between the INS prediction and GPS reading as follows:

(36) $$\left[\begin{array}{c} p_{INS}^{l}-p_{GPS}^{l}\\ vel_{INS}^{l}-vel_{GPS}^{l} \end{array}\right]$$

The *G*, *F*, and *H* matrices in (31) and (32) can be expressed as follows:

(37) $$G_{1\times 15}=\left[\sigma_{p,1\times 3}\;\sigma_{vel,1\times 3}\;\sigma_{\varepsilon ,1\times 3}\;\sigma_{\omega ,1\times 3}\;\sigma_{ax,1\times 3}\right]$$

where *G* is the vector of noise distribution and includes the standard deviation of the state vector $x^{l}$.

(38) $$F_{15\times 15}=\left[\begin{array}{ccccc} 0_{3\times 3} & F_{p} & 0_{3\times 3} & 0_{3\times 3} & 0_{3\times 3}\\ 0_{3\times 3} & 0_{3\times 3} & F_{vel} & 0_{3\times 3} & C_{b}^{l}\\ 0_{3\times 3} & F_{\varepsilon } & 0_{3\times 3} & C_{b}^{l} & 0_{3\times 3}\\ 0_{3\times 3} & 0_{3\times 3} & 0_{3\times 3} & F_{\omega } & 0_{3\times 3}\\ 0_{3\times 3} & 0_{3\times 3} & 0_{3\times 3} & 0_{3\times 3} & F_{ax} \end{array}\right]$$

(39) $$F_{p}=\left[\begin{array}{ccc} \frac{1}{R_{m}+alt} & 0 & 0\\ 0 & \frac{1}{\cos\phi (R_{n}+alt)} & 0\\ 0 & 0 & -1 \end{array}\right]$$

(40) $$F_{vel}=\left[\begin{array}{ccc} 0 & f_{d} & -f_{e}\\ -f_{d} & 0 & f_{n}\\ f_{e} & -f_{n} & 0 \end{array}\right]$$

where $f_{n}$, $f_{e}$, and $f_{d}$ are accelerations of the body along the NED directions; we can transform from ENU to NED and vice versa with the following transformation matrix [54]:

(41) $$C_{NED}^{ENU}=C_{ENU}^{NED}=\left[\begin{array}{ccc} 0 & 1 & 0\\ 1 & 0 & 0\\ 0 & 0 & -1 \end{array}\right]$$

(42) $$F_{\varepsilon }=\left[\begin{array}{ccc} 0 & \frac{-1}{R_{n}+alt} & 0\\ \frac{1}{\left(R_{m}+alt\right)} & 0 & 0\\ 0 & \frac{\tan\phi }{R_{n}+alt} & 0 \end{array}\right]$$

(43) $$F_{ax}=\left[\begin{array}{ccc} -\beta_{ax_{x}} & 0 & 0\\ 0 & -\beta_{ax_{y}} & 0\\ 0 & 0 & -\beta_{ax_{z}} \end{array}\right]$$

(44) $$F_{\omega }=\left[\begin{array}{ccc} -\beta_{\omega_{x}} & 0 & 0\\ 0 & -\beta_{\omega_{y}} & 0\\ 0 & 0 & -\beta_{\omega_{z}} \end{array}\right]$$

The *H* matrix is the design matrix of measurements and, in LC integration, the measurements exactly fit the states of the position and velocity errors; hence,

(45) $$H_{6\times 15}=\left[I_{6\times 6}\;0_{6\times 9}\right]$$

Sequentially, after substitution in Equations (31) and (32), we can obtain the system and measurement LC model, respectively. Finally, the covariance matrix *R*, which has the measured states’ variance in its diagonal, can be expressed as

(46) $$R=diag[\sigma_{\phi }^{2}\;\sigma_{\lambda }^{2}\;\sigma_{alt}^{2}\;\sigma_{vel_{n}}^{2}\;\sigma_{vel_{e}}^{2}\;\sigma_{vel_{d}}^{2}]$$

Also, the prediction covariance matrix $P_{15\times 15}$

(47) $$P_{15\times 15}=diag\left[\sigma_{p,3\times 3}^{2}\;\sigma_{vel,3\times 3}^{2}\;\sigma_{\varepsilon ,3\times 3}^{2}\;\sigma_{\omega ,3\times 3}^{2}\;\sigma_{ax,3\times 3}^{2}\right]$$

#### 3.6.6. TC Integration Model

Unlike LC, in TC integration, the raw measurements of the GNSS receiver (such as pseudo-range and pseudorange-rate) of all MDDA visible satellites are integrated into the sensor fusion system. The system state vector will be [50]

(48) $$\delta x_{17\times 1}^{l}={\left[\delta p_{3\times 1}^{l}\;\delta vel_{3\times 1}^{l}\;\varepsilon_{3\times 1}^{l}\;\delta \omega_{3\times 1}\;\delta ax_{3\times 1}\;\delta b_{r}\;\delta d_{r}\right]}^{T}$$

where $\delta b_{r}\;,\delta d_{r}$ is the receiver clock bias and drift.

Also, the additional part is added to the *F*, *G*, and *H* matrices. Depending on Equation (31), the final system model will be

(49) $$\begin{array}{c} \begin{array}{ccc} & \left[\begin{array}{c} \delta {\dot{p}}_{3\times 1}^{1}\\ \delta {\dot{vel}}_{3\times 1}^{l}\\ {\dot{\varepsilon }}_{3\times 1}^{1}\\ \delta {\dot{\omega }}_{3\times 1}\\ \delta {\dot{ax}}_{3\times 1}\\ \delta {\dot{b}}_{r}\\ \delta {\dot{d}}_{r} \end{array}\right]=\\ & \left[\begin{array}{ccccccc} I_{3\times 3} & F_{p} & 0_{3\times 3} & 0_{3\times 3} & 0_{3\times 3} & 0 & 0\\ 0_{3\times 3} & I_{3\times 3} & F_{vel} & 0_{3\times 3} & C_{b}^{l} & 0 & 0\\ 0_{3\times 3} & F_{e} & I_{3\times 3} & C_{b}^{l} & 0_{3\times 3} & 0 & 0\\ 0_{3\times 3} & 0_{3\times 3} & 0_{3\times 3} & I_{3\times 3}+F_{w} & 0_{3\times 3} & 0 & 0\\ 0_{3\times 3} & 0_{3\times 3} & 0_{3\times 3} & 0_{3\times 3} & I_{3\times 3}+F_{ax} & 0 & 0\\ 0_{1\times 3} & 0_{1\times 3} & 0_{1\times 3} & 0_{1\times 3} & 0_{1\times 3} & 1 & 0\\ 0_{1\times 3} & 0_{1\times 3} & 0_{1\times 3} & 0_{1\times 3} & 0_{1\times 3} & 0 & 1 \end{array}\right]\left[\begin{array}{c} \delta p_{3\times 1}^{l}\\ \delta vel_{3\times 1}^{l}\\ \varepsilon_{3\times 1}^{l}\\ \delta \omega_{3\times 1}\\ \delta ax_{3\times 1}\\ \delta b_{r}\\ \delta d_{r} \end{array}\right]+ & \left[\begin{array}{c} \sigma_{p,3\times 1}\\ \sigma_{vel,3\times 1}\\ \sigma_{z,3\times 1}\\ \sigma_{w,3\times 1}\\ \sigma_{ax,3\times 1}\\ \sigma_{b}\\ \sigma_{d} \end{array}\right]w \end{array} \end{array}$$

where $\sigma_{b},\sigma_{d}$ is the standard deviation for the clock bias and drift, respectively. The crucial component of the GNSS receiver that needs to be evaluated is the pseudorange. Smartphones give all the components for evaluating the pseudorange, but not its value. The period of time it takes for a navigation message to be transmitted from the satellite to the receiver provides the pseudorange between the $m^{th}$ satellite and the GNSS receiver. Pseudorange can be calculated as follows:

(50) $$\rho^{m}=\left[t_{RX}-t_{TX}\right]c$$

where $\rho^{m}$ is the pseudorange from the $m^{th}$ satellite; $t_{TX},t_{RX}$ are the times of transmission and arrival, respectively; and *c* is the speed of light. On the other hand, the pseudorange rate for the $m^{th}$ satellite ${\dot{\rho }}^{m}$ can be determined as

(51) $${\dot{\rho }}^{m}=\frac{-D^{m}c}{L_{1}}$$

where $D^{m}$ is the Doppler shift, which is based on the receiver and satellite motion, and $L_{1}$ is the transmission frequency of the satellite.

(52) $$D^{m}=\frac{\left[\left(vel^{m}-vel\right)\cdot 1^{m}\right]L_{1}}{c}$$

where $vel,vel^{m}$ are the velocity vector of the receiver and the $m^{th}$ satellite in ECEF, respectively, and $1^{m}$ is the line-of-sight (LOS) unit vector from the $m^{th}$ satellite to the GNSS receiver, expressed as

(53) $$1^{m}=\frac{{\left[\left(x-x^{m}\right),\left(y-y^{m}\right),\left(z-z^{m}\right)\right]}^{T}}{\sqrt{\left({\left(x-x^{m}\right)}^{2}+{\left(y-y^{m}\right)}^{2}+{\left(z-z^{m}\right)}^{2}\right)}}$$

where x,y, and *z* are the receiver position and $x^{m},y^{m}$, and $z^{m}$ are the position of the $m^{th}$ satellite in ECEF. The linearization of Equations (50) and (51) leads to the TC measurement model:

(54) $$\left[\begin{array}{c} \delta \rho^{1}\\ \vdots \\ \delta \rho^{m}\\ \delta {\dot{\rho }}^{1}\\ \vdots \\ \delta {\dot{\rho }}^{m} \end{array}\right]={\left[H\right]}_{2m\times 17}\left[\begin{array}{c} \delta p_{3\times 1}^{l}\\ \delta {vel}_{3\times 1}^{l}\\ \varepsilon_{3\times 1}^{l}\\ \delta \omega_{3\times 1}\\ \delta ax_{3\times 1}\\ \delta b_{r}\\ \delta d_{r} \end{array}\right]+\left[\begin{array}{c} v_{\rho }^{1}\\ \vdots \\ v_{\rho }^{m}\\ v_{\dot{\rho }}^{1}\\ \vdots \\ v_{\dot{\rho }}^{m} \end{array}\right]$$

where $\delta \rho^{m}$ and $\delta {\dot{\rho }}^{m}$ are the pseudorange error from the $m^{th}$ satellite and pseudorange rate error, respectively; $v_{\rho }^{m}$ is the effect of residual errors; and $v_{\dot{\rho }}^{m}$ is the observation error.

**Figure 5:**
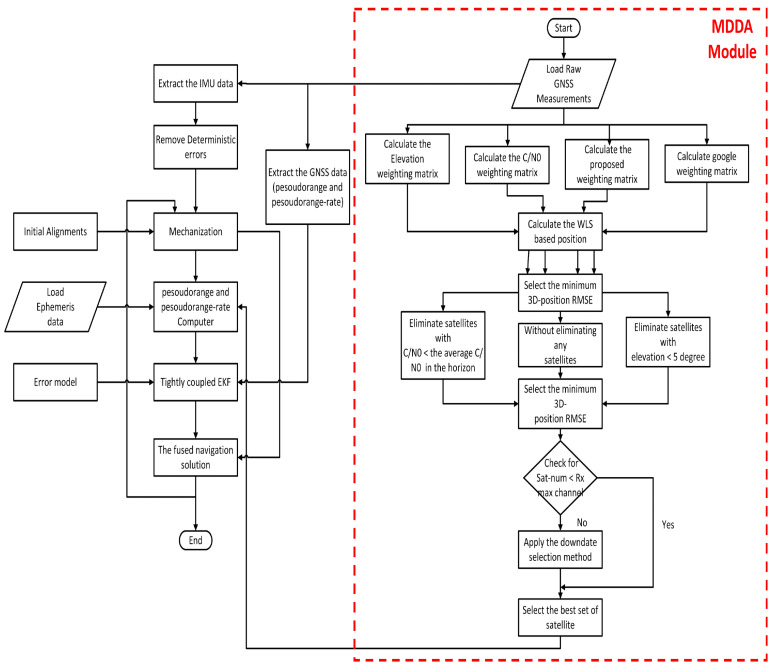
MDDA-TC algorithm flowchart.

The flowchart depicted in Figure 5 provides an overview of the comprehensive MDDA-TC integration process. It commences with the selection of the most suitable weighting matrix that delivers the best 3D position accuracy. Following this, the initial step of the satellite selection strategy is implemented. This entails selecting the satellite with the minimum 3D position error from among three methods: eliminating satellites with $C/N_{o}$ values lower than the average of the $C/N_{o}$ values on the horizon, eliminating satellites with elevation angles less than $5^{\circ }$, or retaining all satellites without elimination.

Subsequently, a check is performed to ascertain if the number of available satellites exceeds the receiver’s channel capacity. If there are more satellites than available channels, the dawndate satellite selection method is applied to identify the best subset. This constitutes the primary segment of the proposed algorithm, hereafter referred to as MDDA.

The following step is that for the TC algorithm, which comes into play after the selection of the optimal satellite set. In this phase, pseudorange computation takes place. The KF is then employed to gauge the discrepancies between GPS pseudo-range and pseudo-range rate measurements and their respective INS-predicted values. These disparities are utilized to estimate the errors inherent to the INS. Subsequently, the output produced by the INS is fine-tuned to accommodate these errors, ultimately yielding an enhanced integrated navigation solution.

## 4. Experimental Setup

**Figure 6:**
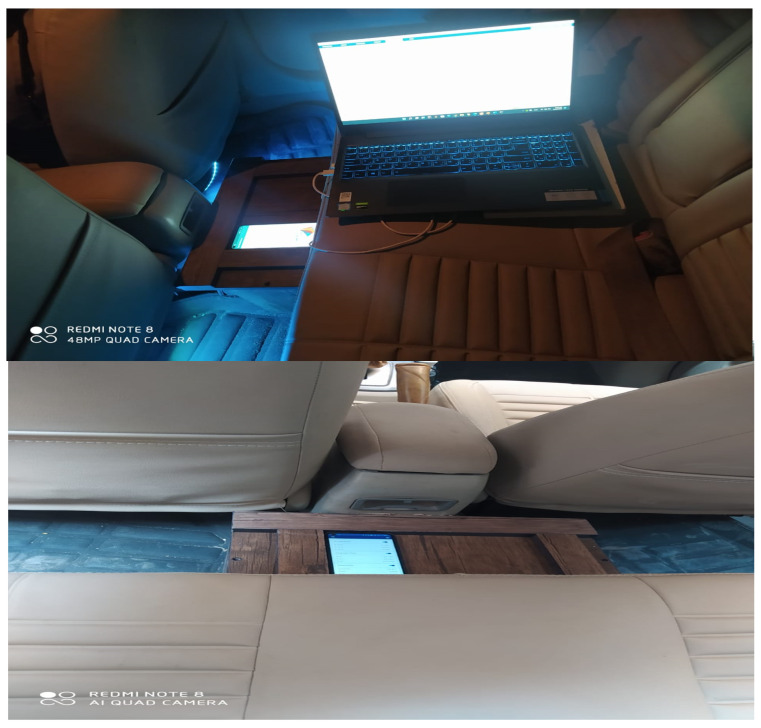
Mounting of the smartphone within the vehicle.

To validate the proposed methodology, a road trajectory was conducted in Cairo, Egypt, within a suburban environment. The smartphone used for the experiment was securely mounted inside the testing land vehicle, as depicted in Figure 6, aligning with the vehicle’s axes. The starting point of the trajectory was carefully selected, considering the initialization of the employed sensors, and the entire trajectory lasted approximately 8 min. The data collection process was carried out using the REALME RMX2030 smartphone, which operates on Android 11 and is equipped with a Qualcomm Snapdragon 665 SM6125 processor. The road test was performed using the fused location provider (FLP) trajectory data as a reference. The FLP system integrates multiple location technologies, including GNSS (multiple constellations), Wi-Fi, and network-based positioning, to provide accurate location information [55,56].

## 5. Results and Discussion

This study explored various scenarios:

1. MDDA was applied to all visible satellites and processed exclusively by the receiver.
2. MDDA was integrated into the LC architecture to enhance its overall performance.
3. MDDA was also incorporated into the TC architecture to further optimize performance.
4. Lastly, all these scenarios were assessed while taking into account the additional constraints posed by restricted receiver channels.

The MDDA algorithm proposed in this study was applied to all visible satellites, resulting in a significant improvement in position accuracy. The evaluation of the algorithm’s performance was based on the comparison of 2D and 3D position root mean square error (RMSE) values. The RMSE was calculated by squaring the differences between the reference solution and the solution obtained from the proposed system and then taking the square root of the mean of these squared errors, as described by Abosekeen et al. [57]. The equation for calculating the RMSE is shown in Equation (55):

(55) $$RMSE=\sqrt{\frac{1}{k}\sum_{k}{\left(p_{k,ref}-p_{k,proposed}\right)}^{2}}$$

where *k* is the time epoch number, $p_{k,ref}$ is the reference position, and $p_{k,proposed}$ is the position for the proposed system.

### 5.1. MDDA with Receiver-Only Scenario

In this scenario, the receiver solution was subjected to the MDDA and then compared with the solution obtained from all visible satellites. The horizontal RMSE improved by 13%, while the 3D position RMSE experienced a significant improvement of 43% over the solution from all visible satellites based on the Google weighting matrix. To further explore the challenges, a low-cost GPS receiver was employed, limiting the maximum number of accessible channels to 10. Instead of utilizing all observed satellites on the horizon, various satellite selection (SS) approaches were examined to assess the algorithm’s performance. The results, presented in Table 2, illustrate the efficacy of the MDDA compared to alternative algorithms. In terms of the 2D position RMSE, the MDDA demonstrated a 12.5% improvement over the EL-based algorithm and a 8.7% improvement over the CNR-based algorithm. Similarly, for the 3D position RMSE, the MDDA showcased a 5.5% improvement over the EL-based algorithm and about 4% improvement over the CNR-based algorithm. It is noteworthy that these improvements were achieved solely utilizing the GPS constellation as the data source.

**Figure 7:**
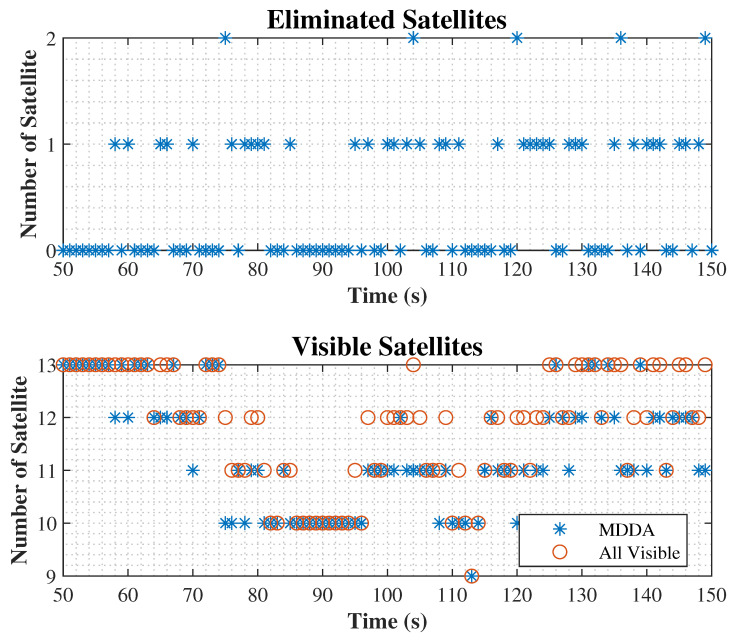
Satellite visibility and elimination.

In Figure 7, the visibility of all satellites and the eliminated satellites using MDDA during samples of epochs are shown.

**Figure 8:**
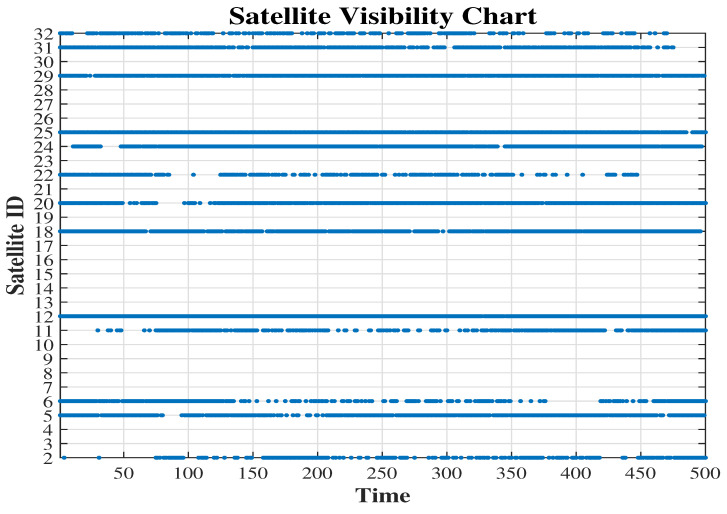
Satellite visibility chart.

In Figure 8, the satellite visibility chart for the trajectory is presented.

### 5.2. MDDA with LC Integration Scenario

In the current scenario, the LC algorithm was employed to improved the position’s RMSE. The MDDA-LC algorithm improves the 2D and 3D position RMSE by 25% and 66.8%, respectively, over the solution from all visible satellites based on the Google weighting matrix in the case of using only the receiver and about 8% improvement in both against the solution from all satellites with LC integration. Additionally, it improved the limited receiver with only 10 channels 10MDDA-LC by 24% in 2D and 40% in 3D position RMSE over 10MDDA with a receiver only, shown in Table 2, and about 18% and 4% in 3D position RMSE over 10EL-LC and 10CNR-LC, respectively. The results are shown in Table 3.

**Figure 9:**
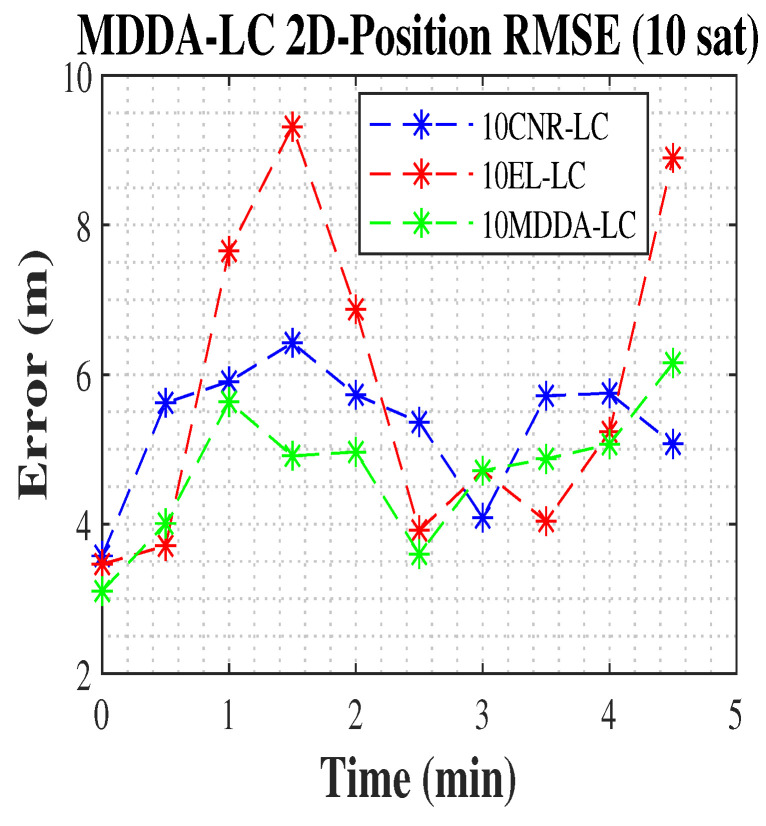
The 2D position RMSE.

**Figure 10:**
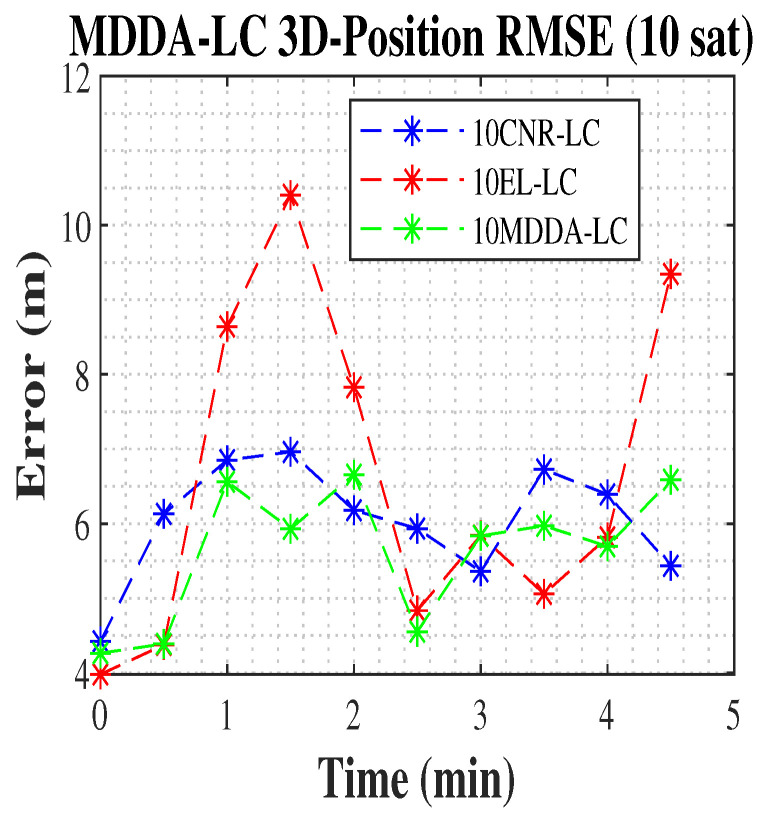
The 3D position RMSE.

In Figure 9 and Figure 10, the 2D and 3D position RMSE values are plotted, with data points sampled at 30-s intervals. The results clearly demonstrate the superior performance of the MDDA-LC algorithm compared to other algorithms, as it consistently achieved the lowest position RMSE.

**Figure 11:**
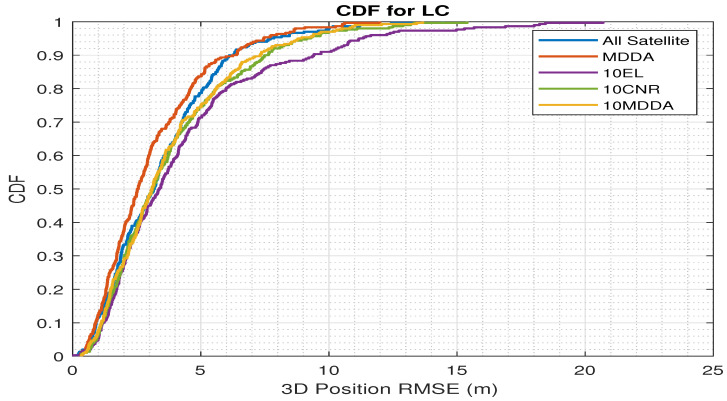
CDF vs 3D-osition RMSE for LC.

Additionally, Figure 11 includes an illustration of the cumulative distribution function (CDF) curve with the 3D position RMSE for LC integration. The comparison indicates that the MDDA, when compared with all satellites on the horizon, exhibited the highest accuracy. Furthermore, when confronted with reducing the number of satellites to match the maximum receiver channels, the MDDA outperformed other techniques, demonstrating superior performance.

### 5.3. MDDA with TC Integration Scenario

In the present scenario, the MDDA-TC algorithm demonstrated a significant enhancement, resulting in an increase that exceeded 75.5% in 2D and 90% in 3D position RMSE over the solution from all visible satellites based on the Google matrix in Table 2, in addition to an improvement of about 78% in 2D and 83% in 3D position RMSE against the MDDA only scenario, and about 2.5% in 2D and 1.7% in 3D position RMSE over the solution from all satellites, shown in Table 4. Additionally, with the limited channel receiver, the 10 MDDA-TC algorithm improved the 2D and 3D position RMSE, with the same improvements over 10 MDDA, shown in Table 2, and about 10% and 4% in 3D position RMSE over 10 EL-TC and 10 CNR-TC, respectively. The results are included in Table 4.

**Figure 12:**
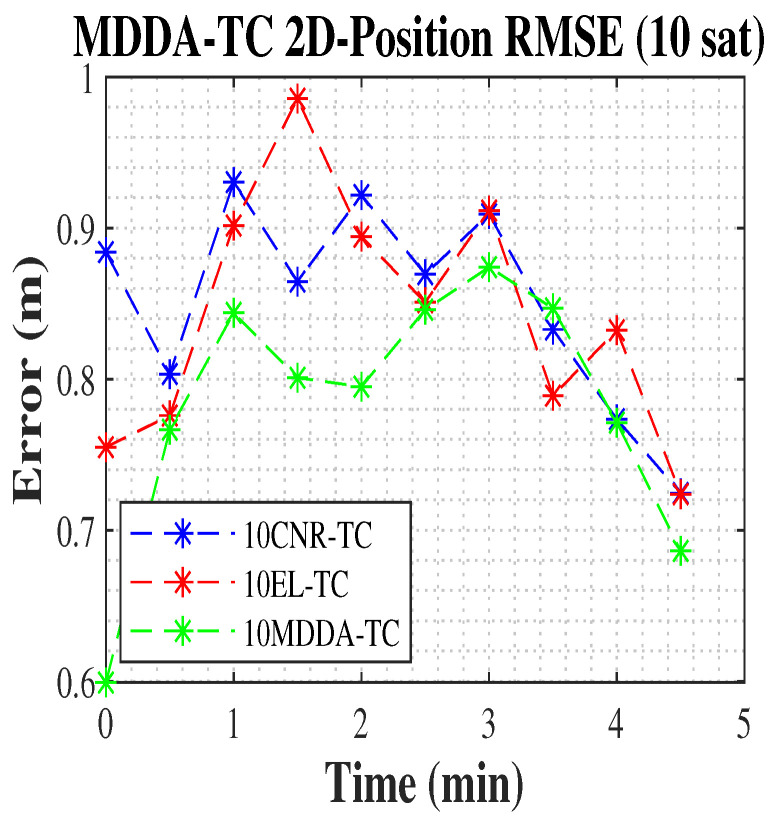
The 2D position RMSE.

**Figure 13:**
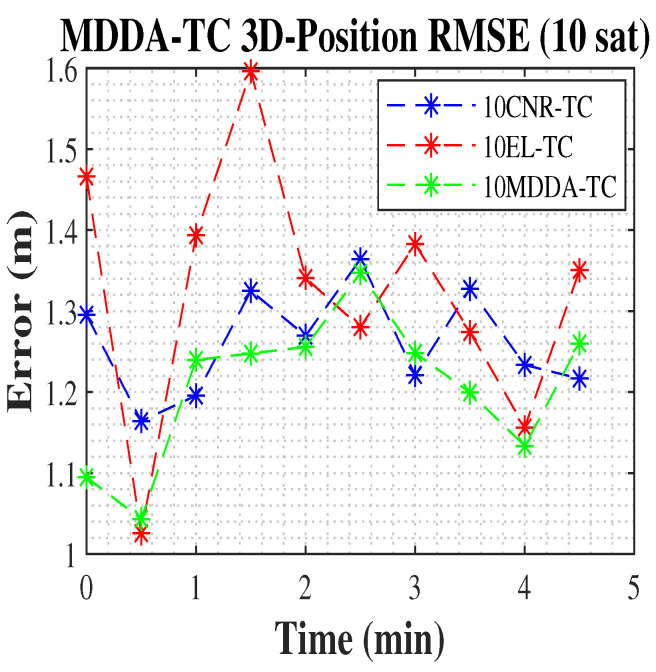
The 3D position RMSE.

Similarly, Figure 12 and Figure 13 demonstrate the improvement in position RMSE achieved by the MDDA-TC algorithm compared to the CNR and EL biased algorithms.

**Figure 14:**
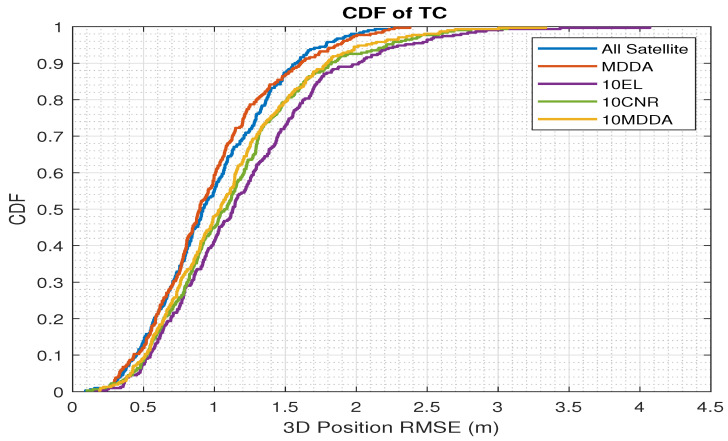
CDF vs. 3D position RMSE for LC.

Furthermore, an additional analysis is depicted in Figure 14, which features the CDF curve in conjunction with the 3D position RMSE for TC integration. The comparison reveals that the MDDA demonstrated optimum accuracy in comparison to all satellites on the horizon. Moreover, in situations requiring the reduction of satellites to align with the maximum number of receiver channels, the MDDA exhibited superior performance compared to alternative methods.

**Figure 15:**
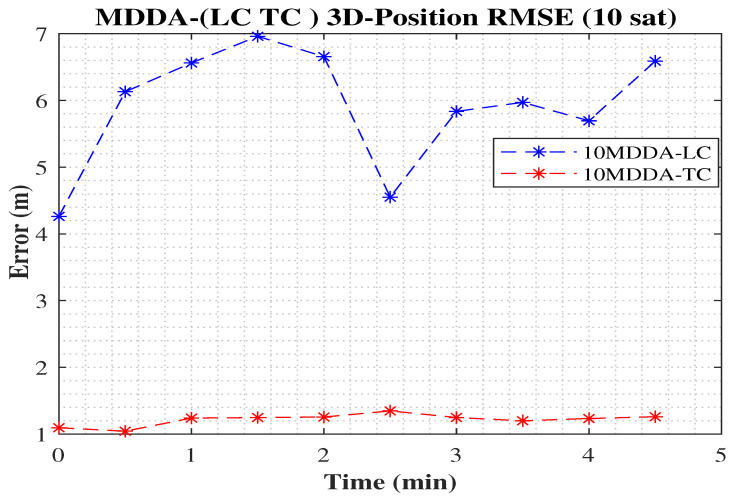
The 3D position error using MDDA-(LC and TC) revealed a noteworthy improvement achieved by utilizing MDDA-TC with a restricted set of 10 visible satellites.

Hence, Figure 15 clarifies the optimization of the MDDA-TC over MDDA-LC algorithms and demonstrates the substantial improvement in the positional RMSE during the whole trajectory.

**Figure 16:**
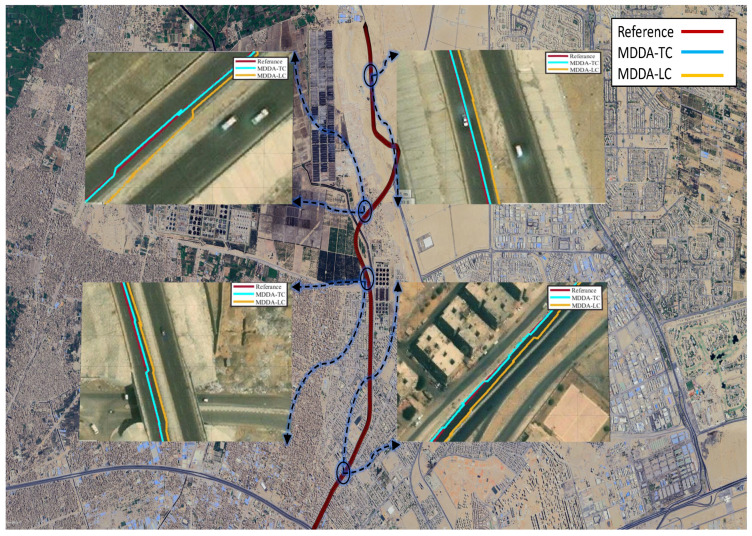
Trajectory depicted on Google earth map.

Figure 16 illustrates the differences between the MDDA-LC and MDDA-TC trajectories in comparison to the reference trajectory, using a real-world map as context. Both methods exhibited satisfactory positioning accuracy, with MDDA-TC demonstrating comparatively enhanced performance.

**Figure 17:**
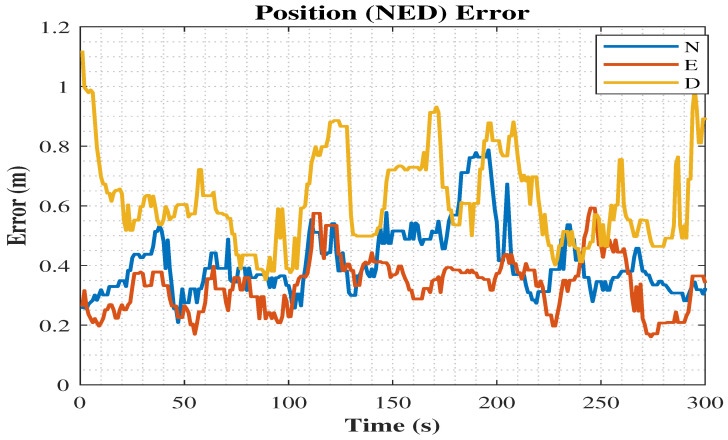
The MDDA-TC position error in (north, east, down) NED.

As shown in Figure 17, the NED position error for the MDDA-TC algorithm, which represented the optimized approach, demonstrated the lowest error compared to the other algorithm.

**Figure 18:**
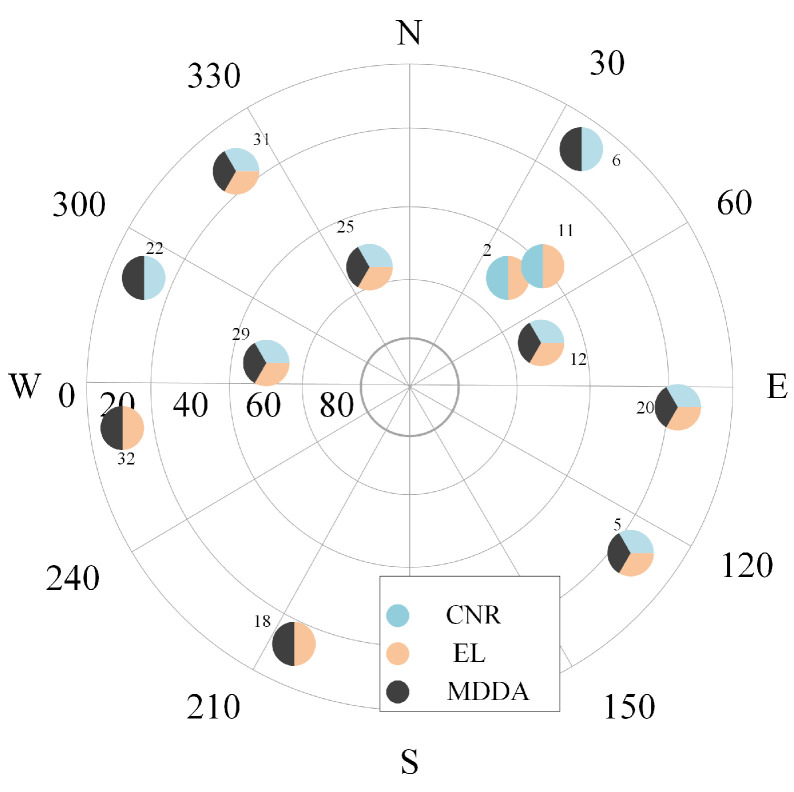
First epoch skyplot.

**Figure 19:**
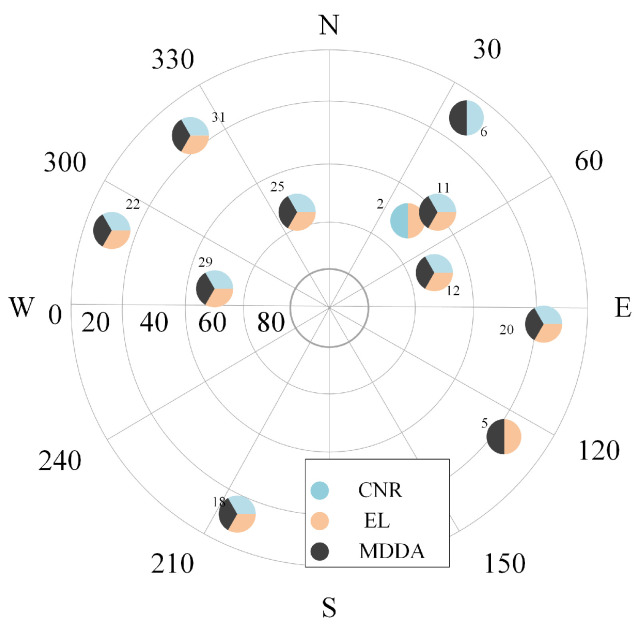
Last epoch skyplot.

Furthermore, an essential question arose, warranting a comprehensive examination: Among the visible satellites, which one was selected by the satellite selection (SS) techniques, and what were the underlying reasons? In order to provide an elucidating response, two distinct epochs are chosen as illustrative examples. The skyplot of the first epoch is presented on the left Figure 18, while the skyplot of the final epoch is displayed on the right in Figure 19. This visual representation facilitates the analysis of the SS techniques and their impact on satellite selection.

**Figure 20:**
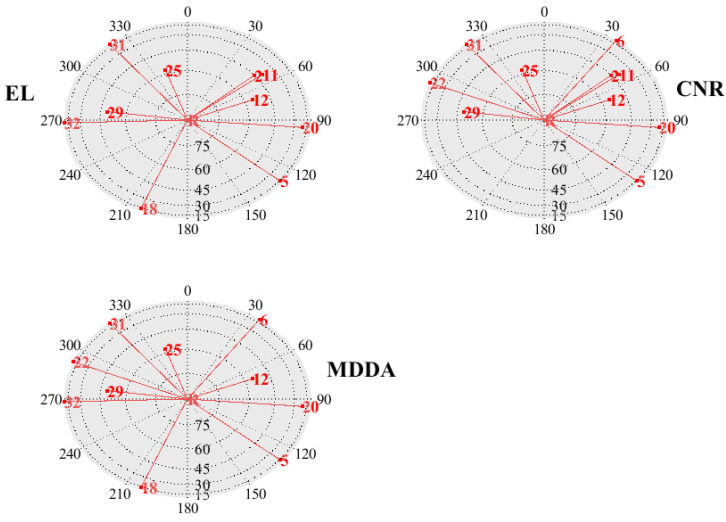
Geometry of the selected satellite.

**Figure 21:**
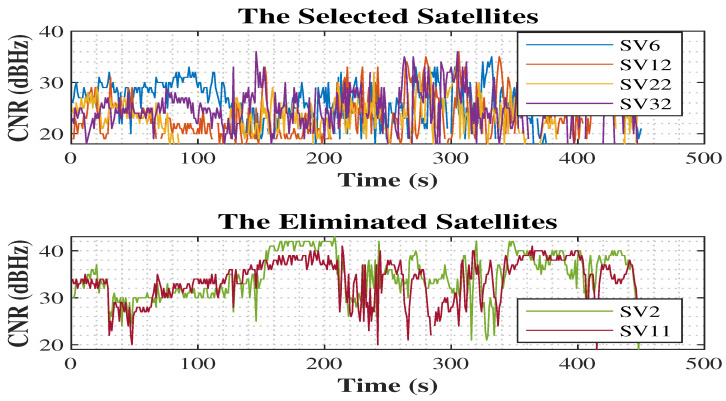
Carrier-to-noise ratio (CNR) of the selected and eliminated satellites.

In the first epoch, the MDDA algorithm prioritized satellites 6, 18, 22, and 32 based on their optimal horizon geometry. However, despite having favorable EL angles and CNR, satellites 2 and 11 were excluded from the selection process. The specific satellite positions and the visualization of this selection process can be observed in Figure 20 and Figure 21, respectively.

The proposed MDDA considered both CNR and El to select the optimal satellite geometry on the horizon during the final epoch. In addition, the average of the dilution of precision (DOP) was computed for the whole trajectory to support the findings and insights regarding the optimal satellite geometry. The results of these analyses are summarized in Table 5.

**Figure 22:**
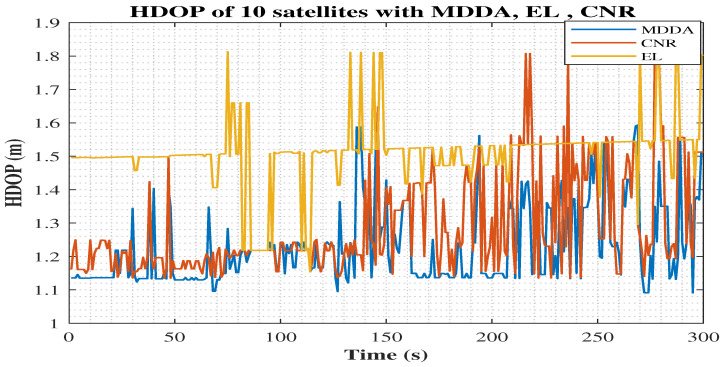
Horizontal dilution of precision (HDOP) for different algorithms.

The graphical representation in Figure 22 provides clear evidence that the MDDA consistently maintained the most favorable geometry throughout the trajectory, as observed in the horizontal dilution of precision (HDOP). The MDDA demonstrated a consistent pattern of superiority, with a lower average HDOP value of 1.28, compared to CNR, with an average HDOP of 1.35, and EL, with an average HDOP of 1.54. This indicated that the MDDA consistently offered superior precision and geometry throughout the trajectory.

**Figure 23:**
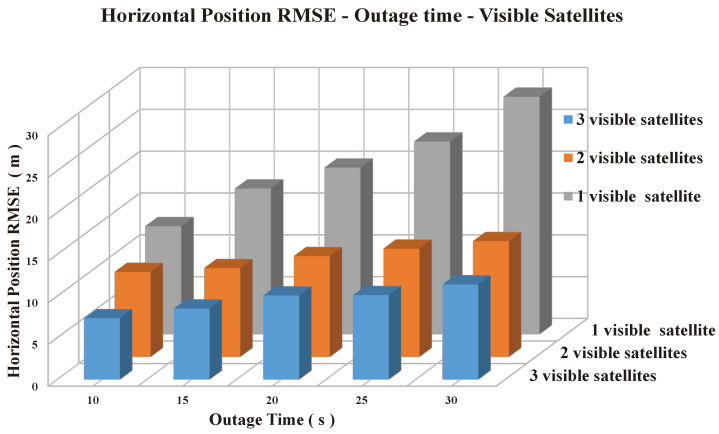
Horizontal position RMSE comparison with respect to the number of available satellites and a simulated outage duration.

Finally, a simulated outage was conducted to assess the performance of the TC algorithm implemented in this study. The outage simulations varied in duration, ranging from 10 to 30 s, and were performed with different numbers of available satellites, from 3 down to 1. The results of these simulations are shown in Figure 23. In comparison, the LC algorithm’s performance deteriorated as the number of available satellites decreased. When fewer than four satellites were available, the LC algorithm was unable to update the solution using GPS measurements, as it depended on previously known GPS position and velocity information. Without GPS updates, the LC approach gradually drifted over time, requiring a new update to correct the error and restore accurate positioning. On the other hand, the performance of the TC integration method remained robust, even during satellite outages, showcasing its capability to maintain accurate positioning with fewer satellites.

## 6. Conclusions

High-precision positioning and navigation have seen significant advancements, with the integration of new technologies aimed at enhancing accuracy and reliability. As these technologies mature, their applications in various domains continue to expand. This study proposes two algorithms for GNSS/INS integration, evaluated using a real-road trajectory to assess the performance enhancement of smartphone sensors. The developed MDDA method was applied to all visible satellites, leading to a 13% improvement in 2D position RMSE and a 43% improvement in 3D position RMSE compared to the solution based on the Google weighting matrix. The LC algorithm was employed to further reduce position RMSE. The combined MDDA-LC algorithm achieved reductions of 25% and 66.8% in 2D and 3D position RMSE, respectively. Meanwhile, the MDDA-TC algorithm demonstrated even greater improvements, exceeding 90% in 2D and 75% in 3D position RMSE compared to the baseline solution. Additionally, the smartphone’s GNSS receiver faced novel constraints, limiting the solution to the most accessible channels rather than utilizing all detected satellites within view. Both the MDDA-LC and MDDA-TC algorithms exhibited equivalent enhancements in 2D and 3D position RMSE, demonstrating their effectiveness in improving positioning accuracy.

## Figures and Tables

**Table 1:** Specifications of MEMS inertial sensors for the used smartphone [48].

	Gyroscope	Accelerometer
Max measurement range	±2000deg/s	±8g
Bias error	±0.4deg/s	±0.6mg
Output noise	$0.007\;\deg/s/\sqrt{\mathrm{Hz}}$	$180\;g/\sqrt{\mathrm{Hz}}$
Resolution	$4.8\times 10^{-4}\;\deg/s$	±0.975mg

**Table 2:** Receiver-only 2D and 3D position RMSE in meters.

Method	$\begin{array}{c} 2D\mathrm{Position} \end{array}$	$\begin{array}{c} 3D\mathrm{Position} \end{array}$
All Visible Satellites		
All Satellites	4.58	11.89
MDDA-Based	3.96	6.78
**Selected Satellites According to Max. Number of Rx Channels (10 Satellites)**		
10 EL-Based	5.67	7.59
10 CNR-Based	5.43	7.40
10 MDDA-Based	4.96	7.17

**Table 3:** MDDA-LC algorithm 2D and 3D position RMSE in meters.

Method	East	North	Down	2D Position	3D Position
**All Visible Satellites**					
All Satellites	3.17	1.98	2.12	3.74	4.30
MDDA-LC-Based	2.86	1.89	1.9	3.43	3.95
**Selected Satellites According to Max. Number of Rx Channels (10 Satellites)**					
10 EL-LC-Based	3.76	2.52	2.41	4.53	5.13
10 CNR-LC-Based	3.19	2.17	2.21	3.86	4.45
10 MDDA-LC-Based	3.18	1.99	2.06	3.75	4.28

**Table 4:** MDDA-TC algorithm 2D and 3D position RMSE in meters.

Method	East	North	Down	2D Position	3D Position
**All Visible Satellites**					
All Satellites	0.66	0.54	0.82	0.86	1.19
MDDA-TC-Based	0.64	0.54	0.82	0.84	1.17
**Selected Satellites According to Max. Number of Rx Channels (10 Satellites)**					
10 EL-TC-Based	0.64	0.73	1.05	0.97	1.43
10 CNR-TC-Based	0.63	0.72	0.94	0.95	1.34
10 MDDA-TC-Based	0.58	0.69	0.92	0.91	1.29

**Table 5:** The average dilution of precision (DOP) error of the whole trajectory.

	EL-Based	CNR-Based	MDDA-Based
Geometric Dilution of Precision (GDOP)	1.90	1.73	1.67
Horizontal Dilution of Precision (HDOP)	1.54	1.35	1.28
Position Dilution of Precision (PDOP)	1.70	1.56	1.50

## Data Availability

Data are contained within the article.
